# Characterization of the expression of LAT1 as a prognostic indicator and a therapeutic target in renal cell carcinoma

**DOI:** 10.1038/s41598-019-53397-7

**Published:** 2019-11-20

**Authors:** Kosuke Higuchi, Shinichi Sakamoto, Keisuke Ando, Maihulan Maimaiti, Nobushige Takeshita, Kentaro Okunushi, Yoshie Reien, Yusuke Imamura, Tomokazu Sazuka, Kazuyoshi Nakamura, Jun Matsushima, Tomomi Furihata, Yuzuru Ikehara, Tomohiko Ichikawa, Naohiko Anzai

**Affiliations:** 1 Department of Pharmacology, Chiba University Graduate School of Medicine, Chiba, Japan; 2 Department of Urology, Chiba University Graduate School of Medicine, Chiba, Japan; 3 Department of Pathology, Dokkyo Medical University Saitama medical center, Saitama, Japan; 4 Department of Clinical Pharmacy and Experimental Therapeutics, Tokyo University of Pharmacy and Life Sciences, Tokyo, Japan; 5 Department of Molecular Tumor Pathology, Chiba University Graduate School of Medicine, Chiba, Japan; 6 Department of Pharmacology and Toxicology, Dokkyo Medical University School of Medicine, Tochigi, Japan

**Keywords:** Renal cell carcinoma, Clinical pharmacology, Prognostic markers, Molecular medicine, Renal cell carcinoma

## Abstract

Large neutral amino acid transporter 1 (LAT1, SLC7A5) is abundantly expressed in various types of cancer, and it has been thought to assist cancer progression through its activity for uptake of neutral amino acids. However, the roles of LAT1 in renal cell carcinoma (RCC) prognosis and treatment remain uncharacterized. Therefore, we first retrospectively examined the LAT1 expression profile and its associations with clinical factors in RCC tissues (n = 92). The results of immunohistochemistry showed that most of the tissues examined (92%) had cancer-associated LAT1 expression. Furthermore, the overall survival (OS) and progression-free survival (PFS) were shorter in patients with high LAT1 expression levels than in those with low LAT1 expression levels (P = 0.018 and 0.014, respectively), and these associations were further strengthened by the results of univariate and multivariate analyses. Next, we tested the effects of JPH203, which is a selective LAT1 inhibitor, on RCC-derived Caki-1 and ACHN cells. It was found that JPH203 inhibited the growth of these cell types in a dose-dependent manner. Moreover, JPH203 clearly suppressed their migration and invasion activities. Thus, our results show that LAT1 has a great potential to become not only a prognosis biomarker but also a therapeutic target in RCC clinical settings.

## Introduction

Renal cell carcinoma (RCC), of which the most prevalent type is clear cell RCC, is a common cancer that accounts for approximately 3.8% of all new cancer cases, and more than 140,000 people worldwide die from RCC every year^1^. RCC has a poor clinical outcome because 20–30% of the patients already have distant metastasis at the time of diagnosis, and 25–40% of RCC patients treated with nephrectomy eventually have relapse or distant diseases^2^. The frequency of 5-year survival rate of metastatic RCC is only 23%^3^.

Two prognostic models have been widely used to obtain clues for prediction of the clinical course and determination of the therapeutic approach for metastatic RCC: the Memorial Sloan Kettering Cancer Center model and the International Metastatic Renal Cell Carcinoma Database Consortium model. However, there is currently no clinically useful prognostic marker for RCC including non-metastatic RCC. Clinicians must therefore determine how to follow-up postoperative patients without any objective guidelines^4^. Therefore, identification of a useful prognostic marker for RCC is needed to improve overall clinical results for RCC.

One of the reasons for the poor survival rate of patients with metastatic RCC has been the lack of effective drugs. However, molecularly targeted drugs and, more recently, immune checkpoint inhibitors have been approved and have contributed to the improvement of prognosis for patients with metastatic RCC^5,6^. Nevertheless, their therapeutic effects are still limited and their side-effects remain difficult issues that often cause treatment failure or require additional therapeutic management^7^. Therefore, the identification of a new therapeutic target that will contribute to the development of a more effective and less toxic treatment strategy for metastatic RCC treatment is also needed.

For the above-described needs for RCC treatment, large neutral amino acid transporter 1 (LAT1, *SLC7A5*) has the potential to offer promising opportunities. LAT1 is a member of the L-type amino acid transporter family (other members being LAT2 [*SLC7A8*], LAT3 [*SLC43A1*], and LAT4 [*SLC43A2*])^8,9^. It has been shown that LAT1 is abundantly expressed in various types of cancer, including non-small cell lung cancer, breast cancer, biliary tract cancer, pancreatic cancer, and prostate cancer, in a cancer-associated manner^10–15^. Cancer cells require essential amino acids for growth and invasion, and LAT1 serves as a primary Na^+^-independent transport system for uptake of neutral amino acids including several essential amino acids^16^. Furthermore, LAT1-mediated leucine uptake is closely linked to the mammalian target of rapamycin (mTOR) signaling pathway, which plays a key role in cell growth, transcription, and translation^17,18^. Consistently, several studies have shown an association of higher LAT1 expression level with poorer prognosis in various types of cancer^12–15^.

JPH203 (KYT0353) has been developed as a first-in-class LAT1-specific inhibitor^19^. JPH203 is a tyrosine analog and thus competitively inhibits LAT1 transporter functions in several types of cancer cells to attenuate their migration/invasion activities and induce apoptosis, which has been thought to be associated with inactivation of the mTOR signaling pathway^20–22^. Moreover, JPH203 has shown significant inhibitory effects on the growth of xenografted human colon and thyroid cancer cells in mice^23,24^, and its efficacy is currently being evaluated in a clinical trial of patients with several types of cancer^25^.

Based on the above-described findings, LAT1 seems to be a promising prognostic biomarker as well as a molecular target in RCC clinical settings. However, the LAT1 expression profiles in RCC patients and the effects of JPH203 on RCC cells have not been characterized. Therefore, in this study, with the aim of clarifying the roles of LAT1 in RCC prognosis and treatment, we retrospectively investigated the LAT1 expression profile and its association with clinical factors in RCC tissues. The effects of JPH203 were also examined using two RCC cell lines.

## Results

### Characterization of the LAT1 expression profile in renal cell carcinoma

**Figure 1:**
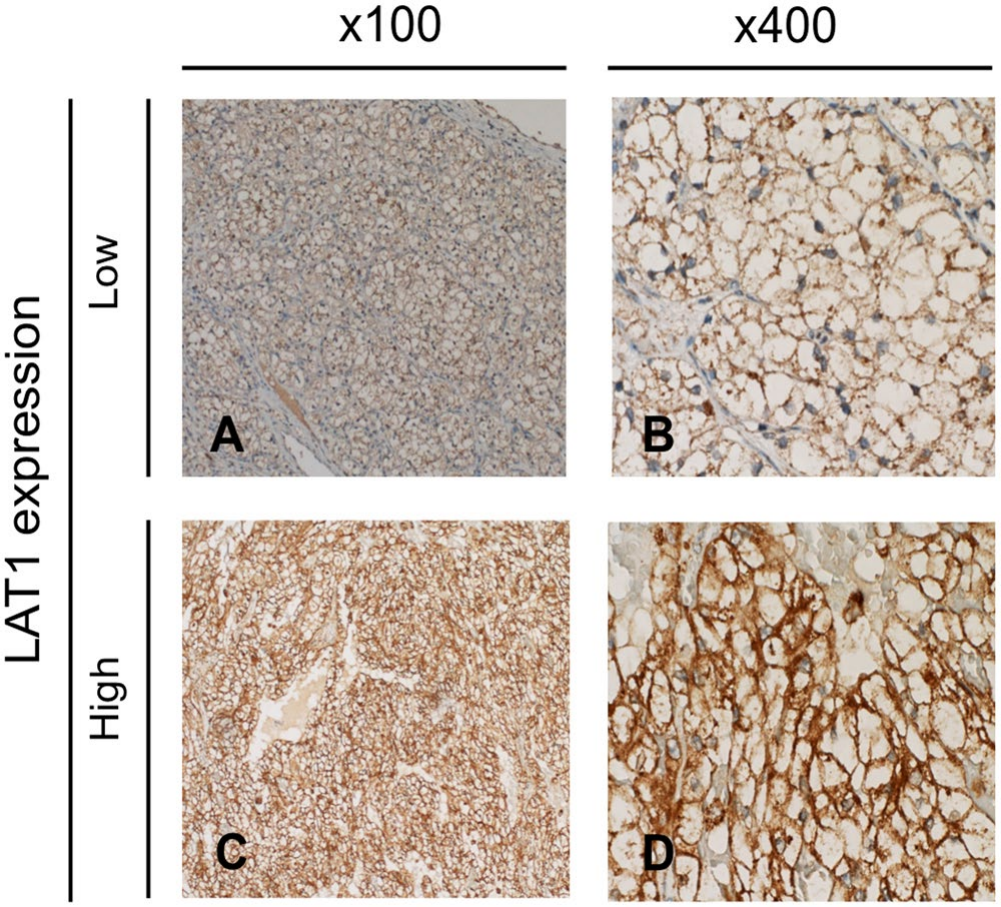
Representative staining pictures showing LAT1 expression in RCC tissues. LAT1 expression in RCC tissues was analyzed by immunohistochemistry. A representative LAT1 immunostaining picture in the low LAT1 expression group is shown at 100-times magnification (panel A) and 400-times magnification (panel B). Similarly, a representative LAT1 immunostaining picture in the high LAT1 expression group is shown at 100-times magnification (panel C) and 400-times magnification (panel D).

In order to characterize the LAT1 expression profile in RCC, we first performed immunohistochemistry using patients’ tissue sections (n = 92). Representative staining pictures of RCC and normal kidney tissues are shown in Figs 1 and S1, respectively. The results showed that 90 patients (97.8%) were LAT1 expression-positive in their cancerous areas, and two patients were negative. The LAT1 staining appeared to be cancer-associated because LAT1 expression in the adjacent normal areas was clearly limited to the proximal tubules, which is a typical LAT1 expression pattern in the human normal kidney as shown in the database (The Human Protein Atlas; https://www.proteinatlas.org).

To investigate whether LAT1 expression levels were associated with clinicopathological factors, we classified the patients into two groups depending on the LAT1 expression scores [S] in their RCC tissues. As shown in Fig. 1, one group was a high LAT1 expression group consisting of 34 patients for whom [S] were 6 to 9 and the other group was a low LAT1 expression group consisting of 58 patients including two negative patients for whom [S] were 0 to 4. Then comparative analyses were conducted to determine relationships of LAT1 expression levels with patients’ age, sex, disease stages, T stages, vascular invasion status, and grades. As shown in Table S1, a high LAT1 expression was correlated with vascular invasion status (P = 0.009) and T stage (P = 0.037).

### Associations of LAT1 expression with overall survival and progression-free survival in renal cell carcinoma

Since LAT1 expression levels were often found in RCC with malignant phenotypes, we examined the associations LAT1 expression with OS and PFS of the patients.

**Figure 2:**
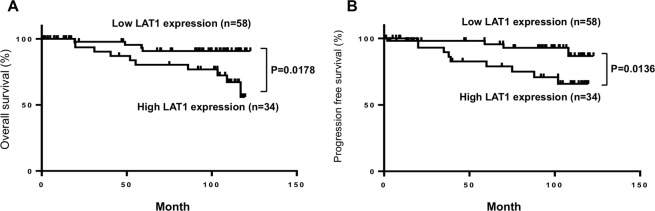
Postoperative survival of RCC patients categorized by LAT1 expression. Cause-specific postoperative survival curves (**A**, overall survival [OS] and **B**, progression free-survival [PFS]) for patients with high and low LAT1 expression levels are shown. Analysis was performed the using Kaplan-Meier method with the log-rank test.

The Kaplan-Meier plots in Fig. 2 showed that OS in the high LAT1 expression group was shorter than that in the low LAT1 expression group (HR: 3.7, 95% CI: 1.3–10.6, P = 0.018). In addition, cause-specific postoperative survival curves for patients with [S] >3 and [S] 0–3 (Fig. S2A), along with those with large (>30%) and small (0–30%) LAT1 expression area (Fig. S2B) are shown. These OS in the high LAT1 expression group were shorter than those in the low LAT1 expression group (HR: 3.4, 95% CI: 1.2–9.5, P = 0.041, and HR: 3.5, 95% CI: 1.2–10.4, P = 0.025, respectively)

Univariate analysis with the Cox proportional hazards model revealed that the LAT1 score and the disease stage, pathological T stage, and histological grade were all significant determinants of OS (Table 1). In multivariate analysis, in addition to the disease stage, the LAT1 score was identified as an independent factor associated with OS (HR: 4.5, 95% CI: 1.1–25.6, P = 0.035) (Table 1).

**Table 1:** Univariable and multivariable cox hazard regression models for OS survival in patients with RCC.

Clinical Factor	Cut off	Univariate Analysis			Multivariate Analysis		
		HR	95% CI	P value	HR	95% CI	P value
Age	70	2.4	0.72–7.31	0.14			
Sex	Male/Female	0.8	0.25–2.8	0.66			
Disease stage	3	7.5	2.57–22.78	0.0003	12.3	1.88–74.8	0.0096
T stage	2	4.9	1.6–14.09	0.0067	2.8	0.41–16.09	0.27
Vascular invasion	2	2.9	0.79–8.66	0.1			
Grade	2	3.7	1.00–23.6	0.05	1.5	0.30–11.12	0.61
LAT1 scare	High/Low	3.5	1.15–12.75	0.026	4.5	1.07–25.55	0.035

Similarly, PFS in the high LAT1 expression group was shorter than that in the low LAT1 expression group in Kaplan-Meier analysis (HR: 3.9, 95% CI: 1.3–12.2, P = 0.014). When univariate analysis was performed, the LAT1 score as well as the disease stage and the histological grade were found to be significant determinants of PFS (Table 2). In multivariate analysis, the LAT1 score and the disease stage were identified as independent factor associated with PFS (HR: 3.7, 95% CI: 1.2–13.4, P = 0.018, and HR: 5.3, 95% CI: 1.8–17.8, P = 0.0033, respectively) (Table 2).

**Table 2:** Univariable and multivariable cox hazard regression models for PFS in patients with RCC.

Clinical Factor	Cut off	Univariate Analysis			Multivariate Analysis		
		HR	95% CI	P value	HR	95% CI	P value
Age	70	1.4	0.37–4.01	0.61			
Sex	Male/Female	0.6	0.21–1.93	0.37			
Disease stage	3	7.8	2.78–22.33	0.0002	5.3	1.76–17.76	0.0033
T stage	2	2.9	0.80–8.56	0.096			
Vascular invasion	2	2.5	0.69–7.33	0.15			
Grade	2	4.2	1.16–27.02	0.026	2.0	0.42–13.79	0.40
LAT1 scare	High/Low	4.4	1.50–15.9	0.0062	3.7	1.24–13.35	0.018

### Functional expression of LAT1 and inhibitory property of JPH203 against LAT1 function in renal cell carcinoma cells

The association found between LAT1 expression and malignant cancer phenotypes suggested that LAT1 has the potential to be a molecular target for RCC therapy, which has remained to be determined in RCC cells. Thus, we aimed to determine whether LAT1 inhibition causes suppression of RCC cells proliferation activity.

**Figure 3:**
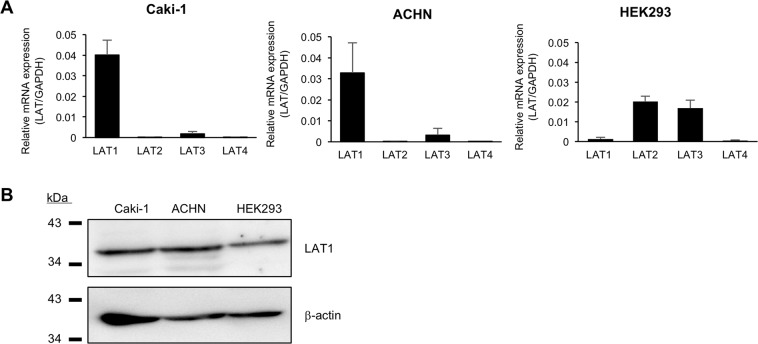
Quantification of mRNA expression levels of LAT family members and detection of LAT1 protein expression in RCC cells. (**A**) LAT1, LAT2, LAT3, and LAT4 mRNA expression levels were analyzed by qPCR in Caki-1, ACHN and HEK293 cells. Experiments were performed at least three times, each performed in duplicate. The mRNA expression levels were normalized to those of β-actin, and data are expressed as means with S.E.M. (**B**) LAT1 protein expression was analyzed by Western blotting in Caki-1, ACHN and HEK293 cells. β-actin was used as a loading control. Representative results from three independent experiments are shown.

First, the functional expression of LAT1 in two representative RCC cell lines, Caki-1 and ACHN cells was examined. Additionally, human embryonic kidney 293 (HEK293) cells were used as comparison purpose. The results of qPCR showed that LAT1 mRNA was clearly expressed in the two cell lines, the levels of which were comparable (Fig. 3A). LAT1 expression was also confirmed by Western blot analysis in these cells (Fig. 3B). In addition to LAT1, the mRNA expression of other LAT family members (LAT2, LAT3, and LAT4) was also detected in the cells. However, their levels were substantially lower than that of LAT1, indicating that LAT1 is exclusively expressed in Caki-1 and ACHN cells. In contrast, LAT1 mRNA expression was quite low in HEK293 cells.

**Figure 4:**
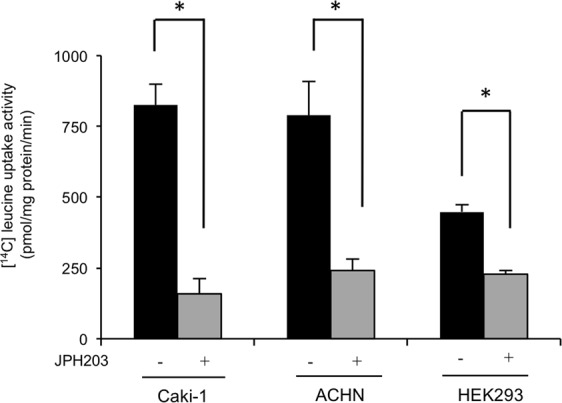
Inhibition of [^14^C]-leucine uptake by JPH203 in RCC cells. The Na^+^-independent [^14^C]-leucine uptake activities (1 µM leucine containing [^14^C]-leucine at 0.1 mCi/mL) of Caki-1, ACHN and HEK293 cells were determined in the presence (+) and absence (−) of JPH203 (10 µM). Determinations were performed three times, each performed in triplicate. Each value represents the mean with S.E.M. *P < 0.05 (unpaired Student’s t-test).

We then tested the effects of JPH203, a selective LAT1 inhibitor, on the system L amino acid transport activity in these cells (Fig. 4). The results of transport assays showed that JPH203 (10 μM) clearly inhibited leucine uptake activities in these cells to decrease their levels to 79% (Caki-1), 68% (ACHN), and 49% (HEK293) of that of the control cells (DMSO-treated cells), respectively.

### Inhibition of growth, migration and invasion inhibition of renal cell carcinoma cells by JPH203 treatment

Since functional LAT1 expression and inhibitory effects of JPH203 on its activity were validated in RCC cells, we assessed the effects of JPH203 on the proliferation activities of these cells.

**Figure 5:**
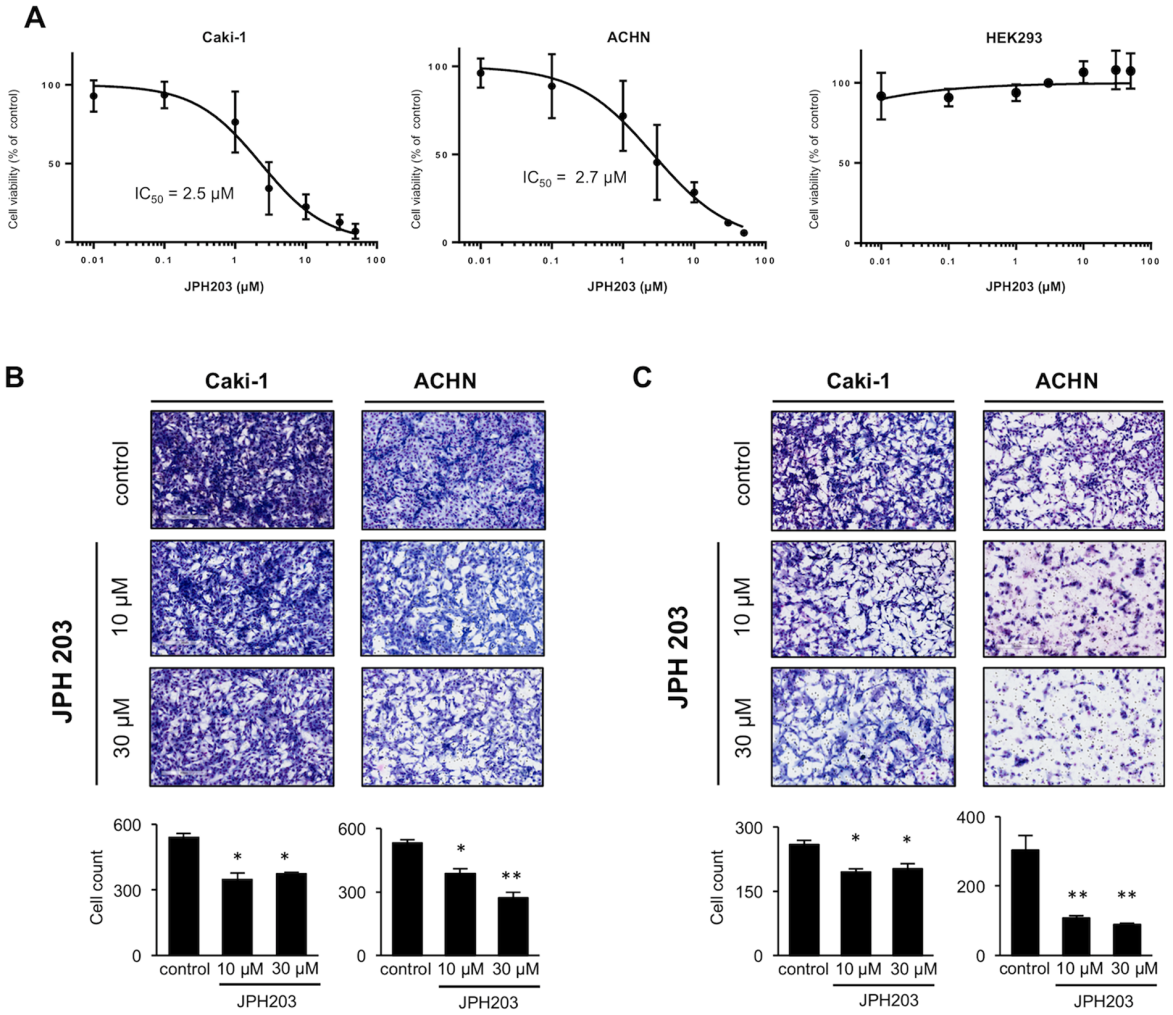
Effects of JPH203 on the viability, migration and invasion activities of RCC cells. (**A**) Caki-1, ACHN and HEK293 cells were treated with various concentrations (0.01, 0.1, 1, 3, 10, 30, and 50 μM) of JPH203 for 96 h. The cell viabilities were determined by WST-8 assays, and viability curves are presented as a percentage of the control value, which was obtained from cells treated with DMSO (0.5%). Data are expressed as means with S.E.M. The *IC*_50_ values for each cell line were calculated using GraphPad Prism 7 J. The experiments were repeated three times, each performed in triplicate. (**B**) Transwell chamber migration assays and (**C**) matrigel invasion assays were used to evaluate the migratory and invasive capabilities of RCC cells following treatment with JPH203 (10 or 30 µM) or DMSO (0.5%) for 48 h. The numbers of cells in five random microscopic fields were counted. Representative images from three independent experiments are shown. Each bar represents the mean with S.E.M. *P < 0.05; **P < 0.01 (unpaired Student’s t-test).

When the cells were treated with JPH203, RCC cells viability were significantly reduced in a dose-dependent manner (Fig. 5A). The IC_50_ values of Caki-1 and ACHN cells were 2.5 and 2.7 μM, respectively, and JPH203 thus showed comparable growth inhibition potencies against the two cell types. In contrast, JPH203 treatment did not show any growth inhibitory effects on HEK293 cells.

We also tested the effects of JPH203 on the migration (Fig. 5B) and invasion (Fig. 5C) abilities of these cells. The results showed that the migration and invasion rates of ACHN cells treated with JPH203 (10 or 30 μM) were significantly lower than those of the control (DMSO-treated) cells (0.7-fold and 0.5-fold lower for migration and 0.4-fold and 0.3-fold lower for invasion, respectively). Similar results were also obtained using Caki-1 cells.

Collectively, the results indicated that JPH203 possessed inhibitory effects on cell proliferation, migration and invasion activities on the RCC cells.

### Inhibition of the mTOR pathway in RCC cells by JPH203

It has been shown that LAT1-mediated leucine uptake stimulates cancer cell proliferation activities through activating the mTOR signaling pathway, which has been considered as a reason why LAT1 functional inhibition reduce cell viability in various cancer cells^18^. Therefore, we presumed that inhibition of the mTOR signaling pathway was also associated with reduction of cell viability of JPH203 in RCC cells.

**Figure 6:**
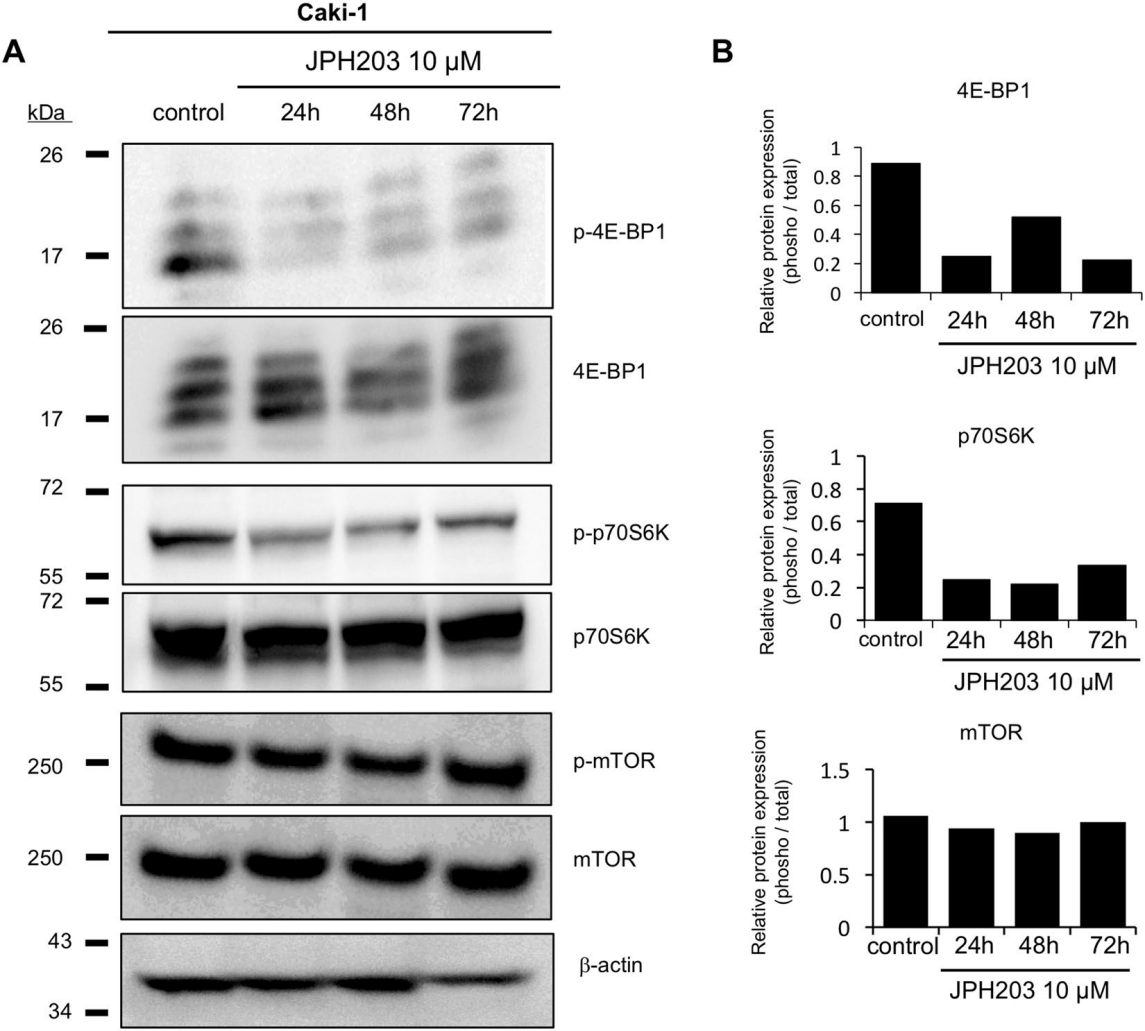
Effects of JPH203 on phosphorylation status of molecules belonging to the mTOR signaling pathway in Cak-1i cells. (**A**) Caki-1 cells were incubated with normal growth medium in the presence of JPH203 (10 µM) for 24 h, 48 h, and 72 h, or DMSO (0.5%, a vehicle control) for 72 h. Whole cellular proteins were extracted and the phosphorylation status of mTOR, p70S6K, and 4E-BP1 was analyzed. β-actin was used as a loading control. Their total protein levels were also examined for references. Representative images from three independent experiments are shown. (**B**) The signal intensities were quantified using the Image J version 1.8.

To examine this possibility, Caki-1 cells were treated with JPH203 (10 μM) for 24, 48, and 72 h to see the phosphorylation status of mTOR and its downstream proteins. As a result, while not so remarkable in mTOR, JPH203 treatment substantially reduced phosphorylation levels of p70S6K and 4E-BP1, indicating that JPH203 inhibited the mTOR signaling pathway in Caki-1 cells (Fig. 6). Similar results were also obtained using ACHN cells (Fig. 3).

To investigate whether inhibition of the mTOR pathway could be involved in pharmacological actions of JPH203, cells were treated with rapamycin (10 or 30 nM) and JPH203 (10 or 30 µM) separately or together for 72 h. Their cell viability of rapamycin-treated cells was significantly reduced, but it did not show any additive or synergistic effects on JPH203 (Fig. S4).

## Discussion

In this study, we clearly showed that LAT1 is overexpressed in RCC in a cancer-associated manner and that inhibition of LAT1 function prevents progression of two RCC cell types. These findings are important advances for future application of LAT1 to RCC diagnosis and treatment.

Regarding the diagnostic property of LAT1, its very high expression frequency in patients with RCC, 97% in 92 patients, is noteworthy. While comparable to that found in gastric cancer (92%, n = 64)^26^, the value is remarkably higher than the values for prostate cancer (24%, n = 54)^27^, lung cancer (36%, n = 160)^28^ and ovarian cancer (39%, n = 142)^29^. Furthermore, the LAT1 detection frequency is also superior to the detection frequency of other genes that are overexpressed in RCC. For example, it has been reported that the PD-L1 expression-positive rate is 36% (n = 453)^30^, and those of vimentin, survivin, and p53 have been reported to be 30% to 80%^31,32^.

In addition, it should be pointed out that the association of LAT1 expression levels with the OS and the PFS in our patients can add further value to LAT1 as a RCC biomarker because identification of a clinically valuable prognostic marker has been needed for RCC. Several proteins have been investigated as potential prognosis biomarkers for RCC. However, their prognostic powers do not seem to be sufficient for use in clinical practice. The mTOR signaling pathway is often activated in RCC, and higher levels of the phosphorylation status of the pathway proteins (mTOR, 4EBP and S6K) have been reported to be associated with poorer prognosis in ccRCC patients (HR = 0.9, 1.5 and 1.5 for OS and HR = 0.9, 2.7 and 2.6 for PFS, respectively)^33^. The expression level of PD-L1 has also been reported to be associated with cancer-specific mortality in RCC patients (HR = 2.0)^30^. However, in comparison to those proteins, LAT1 exhibits high prognostic power (HR = 3.5 and 4.4 for OS and PFS, respectively) at least in our patients.

Collectively, our results strongly suggest that, in line with its high expression frequency, LAT1 is one of the most promising prognostic markers for RCC patients. Thus, it is possible that high levels of LAT1 expression in surgical specimens would indicate the need to follow up the patient closely with frequent imaging in order to take precautions against recurrence and to start treatment as soon as possible Nevertheless, we have to admit that the number of the patients examined in this study is limited, and thus concrete establishment of LAT1 as an RCC prognostic biomarker should await further larger-scale investigations. In addition, we need to explore a way to improve its diagnostic power, possibly by using it in combination with other marker proteins. It has been reported that simultaneous high levels of detection of five different proteins (mTOR, AKT, PTEN, PI3K, and p-4EBP) are more clearly associated with poor prognosis in RCC (HR = 4.8 for OS)^34^. Therefore, it will be worth seeking the best partners for LAT1 to mutually enhance their biomarker potentials in future studies.

Aside from its biomarker potential, our results also indicate the possibility that JPH203-mediated functional LAT1 inhibition is a promising approach for treatment of recurrence of RCC. Of note, since a high expression level of LAT1 is associated with poor prognosis in RCC, an LAT1-targeting anti-cancer approach has the potential to be a key therapeutic option to for malignant RCC.

The effects of JPH203 have been tested in various types of solid cancer, but this report is the first report for RCC cells. It should be mentioned that the efficacy levels in RCC cells (IC_50_ = 2.5 and 2.7 µM) are higher than those reported so far in other cancer types (2.3 to 41.7 µM)^24,35^. Therefore, it can be assumed that RCC is a preferable target for JPH203-mediated therapy. Regarding its mechanisms, it has been considered that JPH203 blocks LAT1-mediated essential amino acid uptake, not only depleting the materials for protein synthesis but also inactivating the mTOR signaling pathway^15,20,23,36,37^. The suppression of phosphorylation levels in mTOR downstream proteins has been validated in our JPH203-treated RCC cells, suggesting that similar mechanisms of action underlie the effects of JPH203. Likewise, lack of additional effects of rapamycin on JPH203 in this study implies that these two agents share the same cellular pathways to exert their pharmacological actions.

Furthermore, considering that LAT1 is overexpressed in cancer cells, LAT1-targeting RCC therapy may offer favorable tolerability. This may be too speculative at present, but in support of this, preliminary results of the first-in-human clinical trials (n = 17, colon, pancreas, bile duct, esophagus, and breast cancer patients) show not only long-term survival for more than two years in a patient with bile duct cancer but also the occurrence of grade 3 or higher side effects (liver damage) in only 12% of the patients^25^. Since side effects have often caused dose delays or reductions of molecularly targeted therapeutics or immune checkpoint inhibitors in RCC patients, it will be important to focus on the safety potential of JPH203 in future studies.

In conclusion, we showed that LAT1 is overexpressed in RCC at a high frequency and that there is an unequivocal association of higher levels of LAT1 expression with poorer prognosis of RCC. We also clarified that functional LAT1 inhibition by JPH203 significantly reduce the proliferation activities of RCC cells. Therefore, LAT1 holds the great potentials not only as a prognosis biomarker but also as a therapeutic target in RCC clinical settings. To further advance our research, we are currently planning to conduct a phase 2 clinical trial for patients with RCC.

## Methods

### Clinical RCC specimens

A total of 92 clinical specimens were obtained from clear cell RCC patients who had undergone nephrectomy or partial nephrectomy at Chiba University Hospital (Chiba, Japan) between April 2007 and March 2011. All of the patients provided the signed informed consent, and the study protocol was approved by the Institutional Review Board of Chiba University (approval no. 484). All experiments were performed in accordance with relevant guidelines and regulations as described by our institution. Information on patients is summarized in Table S2.

### Immunohistochemistry

Immunohistochemical staining for detection of LAT1 was performed on formalin-fixed and paraffin-embedded tissue sections (4 µm in thickness) using a mouse anti-LAT1 monoclonal antibody (clone no. 4D9; Transgenic, Kumamoto, Japan). The detailed experimental methods were reported previously^38^.

### Scoring of immunohistochemical staining

Immunoreactivity for LAT1 was evaluated according to Sinicrope’s method^39^ with minor modifications as reported previously^26^. Briefly, LAT1 expression score [S] was determined on the basis of both the staining intensity level [I] at the carcinoma cell membranes and the staining area level [A], which represents a percentage of the whole carcinoma area, relative to the total section area. [I] was classified as follows: 0, no staining; 1, weakly; 2, moderate; and 3, intense. [A] was classified as follows: 0, none; 1, 1–10% (focal); 2, 11–30% (partial); and 3, >30% (diffuse). [S] for each patient was obtained by multiplying [I] by [A]. Scoring calculations were performed by two independent investigators (J.M. and K.H.) who were blinded to each patients’ clinical status. Based on [S], patients were divided into high and low LAT1 expression groups ([S] 0–4 and [S] 6–9, respectively).

The cut-off value used in Fig. 2 was determined using the Receiver Operating Characteristic (ROC) curve. In addition, other cut-off value [S] 4 or staining area >30%, which were used in Fig. S2, were determined using the respective median value.

### Cell culture

Human RCC cell lines, Caki-1 and ACHN, were obtained from the Cell Resource Center for Biomedical Research, Institute of Development, Aging and Cancer, Tohoku University (Miyagi, Japan). Both cell types were cultured in RPMI-1640 medium supplemented with 10% fetal bovine serum and penicillin-streptomycin. HEK293 cells was cultured in DMEM medium supplemented with 10% fetal bovine serum and penicillin-streptomycin. These cells were maintained in an incubator with a humidified atmosphere of 95% air and 5% CO_2_ at 37 °C.

### Quantitative PCR (qPCR)

The mRNA expression levels of LAT1, LAT2, LAT3, and LAT4 in RCC cells and HEK293 cells were quantified by qPCR. The β-actin mRNA levels were also quantified for use as a normalization control. Experiments were performed according to a protocol reported previously^38^. Information on the specific primers is shown in Table S3.

### Cell viability assay

Inhibitory effects of JPH203 (MedKoo, Morrisville, NC) on the proliferation activities of Caki-1, ACTN and HEK293 cells were evaluated using a WST-8 kit (Nacalai Tesque, Kyoto, Japan) according to the manufacturer’s protocol. The experiment was performed as previously reported^35^. *IC*_50_, which is the concentration at which the cell viability is reduced by half, was calculated using GraphPad Prism 7J (GraphPad Software, La Jolla, CA). Similarly, inhibitory effects of JPH203 and rapamycin (FUJIFILM Wako Pure Chemical Corporation, Osaka, Japan) on the proliferation activities of Caki-1 and ACTN cells were also evaluated using a WST-8 kit. The cells were seeded onto 96-well plates (0.5 × 10^4^ cells/well) and cultured with JPH203 (10 or 30 µM), rapamycin (10 or 30 nM), or DMSO (0.5%, a vehicle control), for three days, after which a viability assay was performed.

### Migration and invasion

Cell migration and invasion were assessed using Corning Falcon Cell Culture Inserts (Thermo Fisher Scientific, Waltham, MA) and Corning BioCoat Matrigel Invasion Chamber (Thermo Fisher Scientific), respectively. The essential parts of the experiments were described previously^40^. The cells were seeded at the upper chamber of a transwell insert at 1 × 10^5^ cells/well and then incubated for 48 hours with serum-free RPMI1640 medium containing JPH203 (10 and 30 µM) or dimethyl sulfoxide (DMSO) for experimental and control groups, respectively. The numbers of cells that had migrated or invaded in five random microscopic fields, which were randomly selected, were counted using Image J version 1.8.0. (National Institutes of Health, Bethesda, MD).

### L-leucine uptake assays

LAT1 function and the ability of JPH203 to inhibit LAT1 function in RCC cells and HEK293 cells were examined by transport assays using L-leucine (leucine) (containing [^14^C]leucine at 0.1 mCi/mL) (PerkinElmer, Boston, MA) as a substrate. These cells were seeded onto 24-well plates (0.5 × 10^5^ cells/well) and cultured for two days, after which a transport assay was performed. The experiment was performed as previously reported^35^.

### Western blot analysis

The cells were dissolved in a sample buffer (25% glycerol, 1% sodium dodecyl sulfate [SDS], 62.5 mM Tris-Cl, 10 mM dithiothreitol) and pre-incubated at 95 °C for 5 min. Aliquots of samples containing 20 µg protein were applied to 10% SDS-polyacrylamide electrophoresis (PAGE) and then transferred onto PVDF membranes. The membranes were incubated at room temperature for 1 hour in TBS-T (10 mM Tris–HCl, 100 mM NaCl, 0.1% Tween-20, pH 7.5) with 5% skim milk (for LAT1 and β-actin) or Blocking One-P (Nacalai Tesque) (for mTOR, phosphorylated mTOR [p-mTOR], 4E-binding protein 1 [4E-BP-1], p-4E-BP-1, ribosomal protein S6kinase beta-1 [p70S6K], and p-p70S6K), after which they were incubated with primary antibodies at 4 °C overnight. After washed with TBS-T, the blots were incubated with an HRP-conjugated anti-rabbit IgG antibody for 1 hour at room temperature. Information on the antibodies used in this study is given in Table S4.

Signals were generated by Clarity MAX Western ECL Substrate (Bio-Rad Laboratories, Hercules, CA) and visualized with LAS-4000 mini (Fujifilm, Tokyo, Japan), of which intensities were quantified using the Image J version 1.8.0.

### Statistical analysis

The Kaplan-Meier method and the log-rank test were used to analyze survival differences. Univariate and multivariate Cox proportional models were used in statistical analyses for association of clinical factors with overall survival (OS) and progression-free survival (PFS). OS was determined using the period from the operation to death for each patient. PFS was defined as the period from the operation to the first disease progression or death. The Wilcoxon signed-rank test and chi-square test were used to assess associations of LAT1 expression with clinical factors. The unpaired Student’s t-test was used for investigating differences between two groups. Statistical calculations were done with JMP Pro, version 13.0.0. (SAS Institute, Cary, NC). P values below 0.05 indicate statistically significant differences.

## Supplementary information

Supplementary information
