# Supplementary material for: Characterization of the expression of LAT1 as a prognostic indicator and a therapeutic target in renal cell carcinoma
Source: Sci Rep. 2019 Nov 20;9:16776. doi: 10.1038/s41598-019-53397-7 (PMC6868143; doi:10.1038/s41598-019-53397-7)

Supplementary information

Characterization of the expression of LAT1 as a prognostic indicator and a therapeutic target in renal cell carcinoma

Kosuke Higuchi [1, 2], Shinichi Sakamoto [2], Keisuke Ando [1, 2], Maihulan Maimaiti [2], Nobushige Takeshita [1, 2], Kentaro Okunushi [1], Yoshie Reien [1], Yusuke Imamura [2], Tomokazu Sazuka [2], Kazuyoshi Nakamura[2], Jun Matsushima [3], Tomomi Furihata [4], Yuzuru Ikehara [5], Tomohiko Ichikawa [2], *Naohiko Anzai [1, 6]

[1] *Department of Pharmacology, Chiba University Graduate School of Medicine, Chiba, Japan*

[2] *Department of Urology, Chiba University Graduate School of Medicine, Chiba, Japan*

[3] *Department of Pathology, Dokkyo Medical University Saitama medical center, Saitama, Japan*

[4] *Department of Clinical Pharmacy and Experimental Therapeutics Tokyo University of*

Pharmacy and Life Sciences, Tokyo, Japan

[5] *Department of Molecular Tumor Pathology, Chiba University Graduate School of Medicine, Chiba, Japan*

[6] *Department of Pharmacology and Toxicology, Dokkyo Medical University School of Medicine, Tochigi, Japan*

Correspondence to: Naohiko Anzai, M. D., Ph. D, Department of Pharmacology, Chiba University Graduate School of Medicine, 1-8-1 Inohana, Chuo-ku, Chiba 260-8670, Japan. Tel/Fax: 043-226-2050/2052, e-mail: anzai@chiba-u.jp

Table S1. Clinical factors associated with LAT1 expression levels in patients with RCC.

Clinical Factor	Low LAT1 (n=34)	High LAT1 (n=58)	P value
Age (AV± SD)	58.6 ± 11.5	62.5 ± 13.4	0.133
Sex (% male)	80.7%	77.1%	0.562
Disease stage (% of stage 3 or greater)	12.2%	20%	0.172
T stage (% of pT2 or greater)	7%	22.8%	0.037
Vascular invasion	5.40%	25%	0.009
Grade (% of grade 2 or greater)	63%	67%	0.428

Table S2. Patients' characteristics

Patients characteristics		number
Age (AV± SD)		58.6 ± 11.5
Sex	male	73
	female	19
Disease stage	1	75
	2	3
	3	6
	4	8
T stage	1	79
	2	4
	3	8
	4	1
Vascular invasion		12
Grade	1	33
	2	51
	3	8
Total nephrectomy		58
Partial nephrectomy		36
LAT1 score	0	2
	1	2
	2	8
	3	34
	4	12
	6	28
	9	6
Follow-up duration (Month, AV± SD)		77.4 ± 40.5

Table S3. Primers

mRNA		Primer (5’ > 3’)
LAT1	forward	TGTACGTGCTGACCAACCTG
	reverse	ATGACGCCCAGGTGATAGTTC
LAT2	forward	CACGGTTGCTGGACAGATAG
	reverse	GGGAACAGCAGGTTGATCTT
LAT3	forward	ATGGACTGGCGGATCAAGG
	reverse	TCTTGCAGTAGCGTGGTCTGATG
LAT4	forward	GGTGGACAAGTTTCTGCTGAGTG
	reverse	CAGCTGTATGAGGATGCGGTGTA
β-actin	forward	CCAACCGCGAGAAGATGA
	reverse	CCAGAGGCGTACAGGGATAG

Table S4. Antibodies

Antibody	Supplier	Dilution
Mouse anti-LAT1 antibody	Trans Genic Inc.	1:200
Rabbit anti-β-actin antibody	Cell Signaling Technology	1:1000
Rabbit anti-mTOR antibody	Cell Signaling Technology	1:1000
Rabbit anti-p-mTOR antibody	Cell Signaling Technology	1:1000
Rabbit anti-4E-BP1 antibody	Cell Signaling Technology	1:1000
Rabbit anti-p-4E-BP-1 antibody	Cell Signaling Technology	1:1000
Rabbit anti-p70S6K antibody	Thermo Fisher Scientific	1:1000
Rabbit anti-p-p70S6K antibody	Cell Signaling Technology	1:1000
ECL-anti-rabbit IgG, horseradish peroxidase linked whole antibody from donkey	GE Healthcare UK Ltd	1:10000
ECL-anti-mouse IgG, horseradish peroxidase linked whole antibody from sheep	GE Healthcare UK Ltd	1:20000

Fig. S1. Representative staining pictures showing LAT1 expression in normal kidney tissues

LAT1 expression in normal kidney tissues was analyzed by immunohistochemistry (A, ×100 B, ×400). Representative staining images are shown.

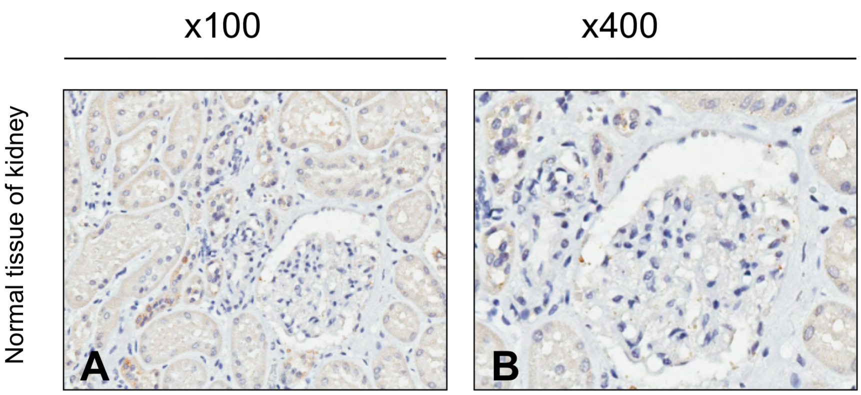

Fig. S2. Postoperative survival of RCC patients categorized by various cutoffs of LAT1 expression

Cause-specific postoperative overall survival curves for patients (A) with [S] 4-9 and [S] 0-3, (B) with >30% of LAT1 expression area and 0-30% of LAT1 expression area are shown. Analysis was performed the using Kaplan-Meier method with the log-rank test.

A

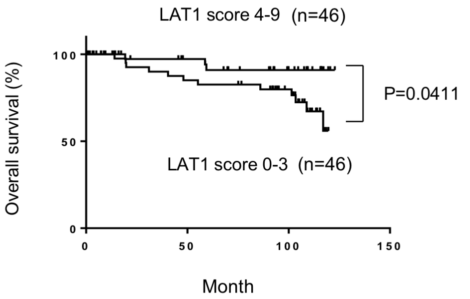

B

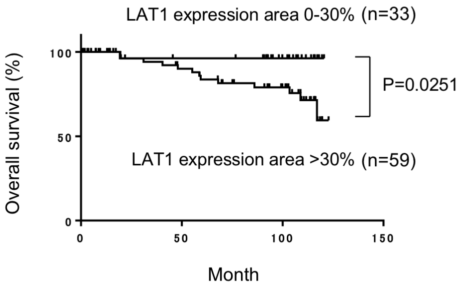

Fig. S3. Effects of JPH203 on phosphorylation status of molecules belonging to the mTOR signaling pathway in ACHN cells

(A) ACHN cells were incubated with normal growth medium in the presence of JPH203 (10 μM) for 24 h, 48 h, and 72 h, or DMSO (0.5%, a vehicle control) for 72 h. Whole cellular proteins were extracted and the phosphorylation status of mTOR, p70S6K, and 4E-BP1 was analyzed. β-actin was used as a loading control. Their total protein levels were also examined for references. Representative images from three independent experiments are shown. (B) The signal intensities were quantified using the Image J version 1.8.0.

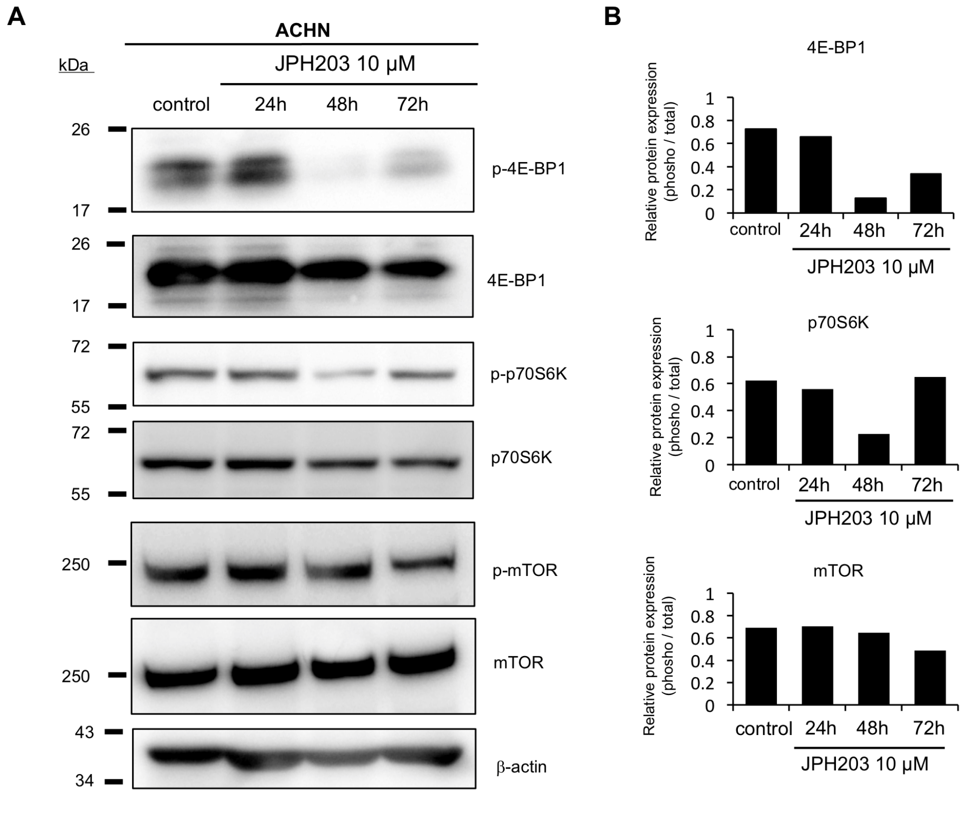

Fig. S4. Effects of rapamycin treated with JPH203 on the viability of RCC cells

Caki-1 and ACHN cells were treated with rapamycin (10 or 30 nM or absence) and JPH203 (10 or 30 μM) or DMSO. The cell viabilities were determined by WST-8 assays, and viability bar graphs are presented as a percentage of the control value, which was obtained from cells treated with DMSO (0.5%). The experiments were repeated three times, each performed in triplicate. Each bar represents the mean with S.E.M. *, P<0.05; n.s. not significant (unpaired Student's t-test).

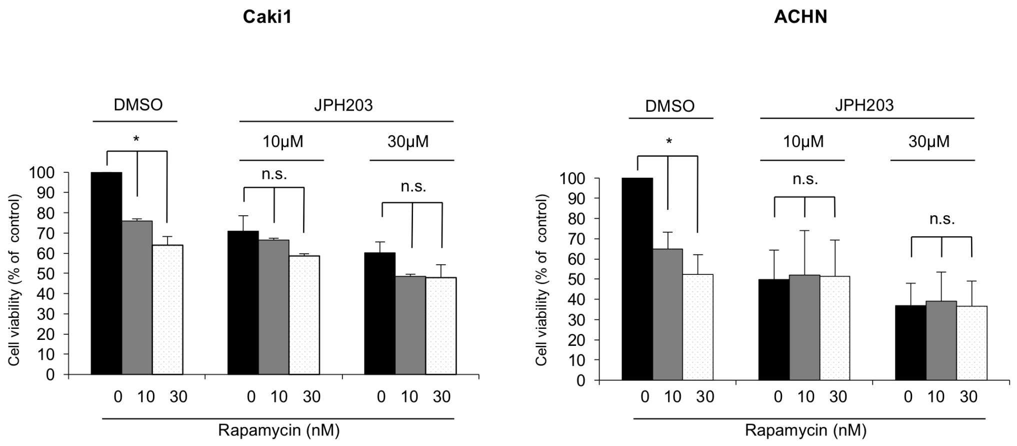

Fig. S5. LAT1and β-actin full-length western blot analysis

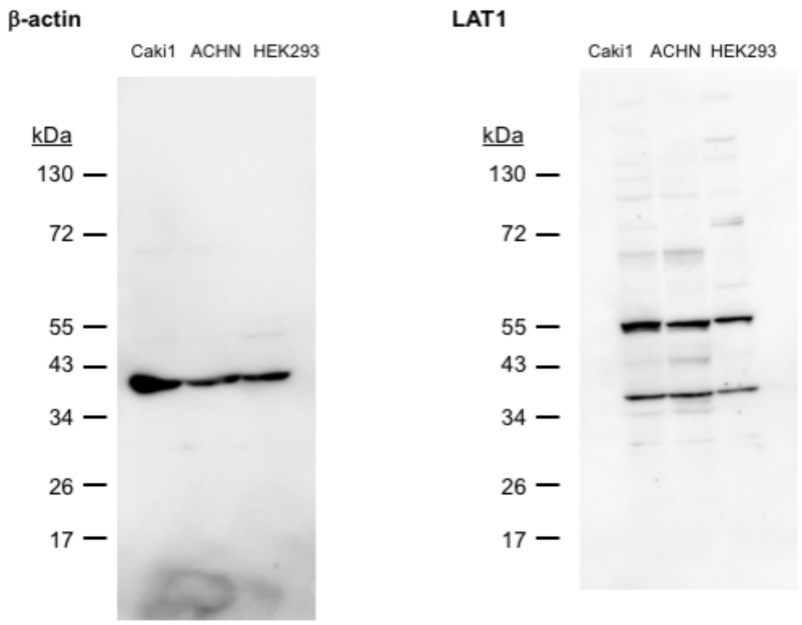

Fig. S6. 4E-BP1and phosphorylated 4E-BP1 full-length western blot analysis

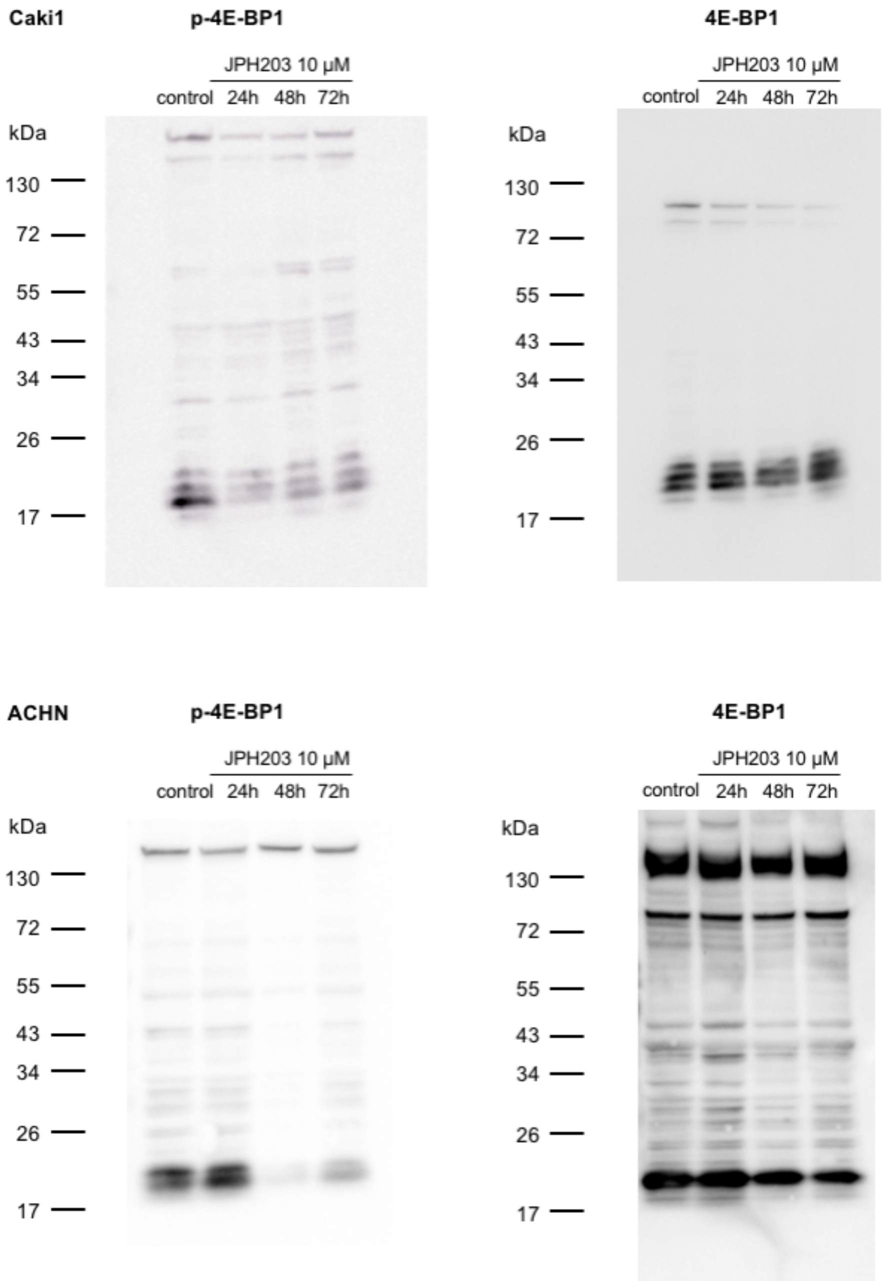

Fig. S7. p70S6K and phosphorylated p70S6K full-length western blot analysis

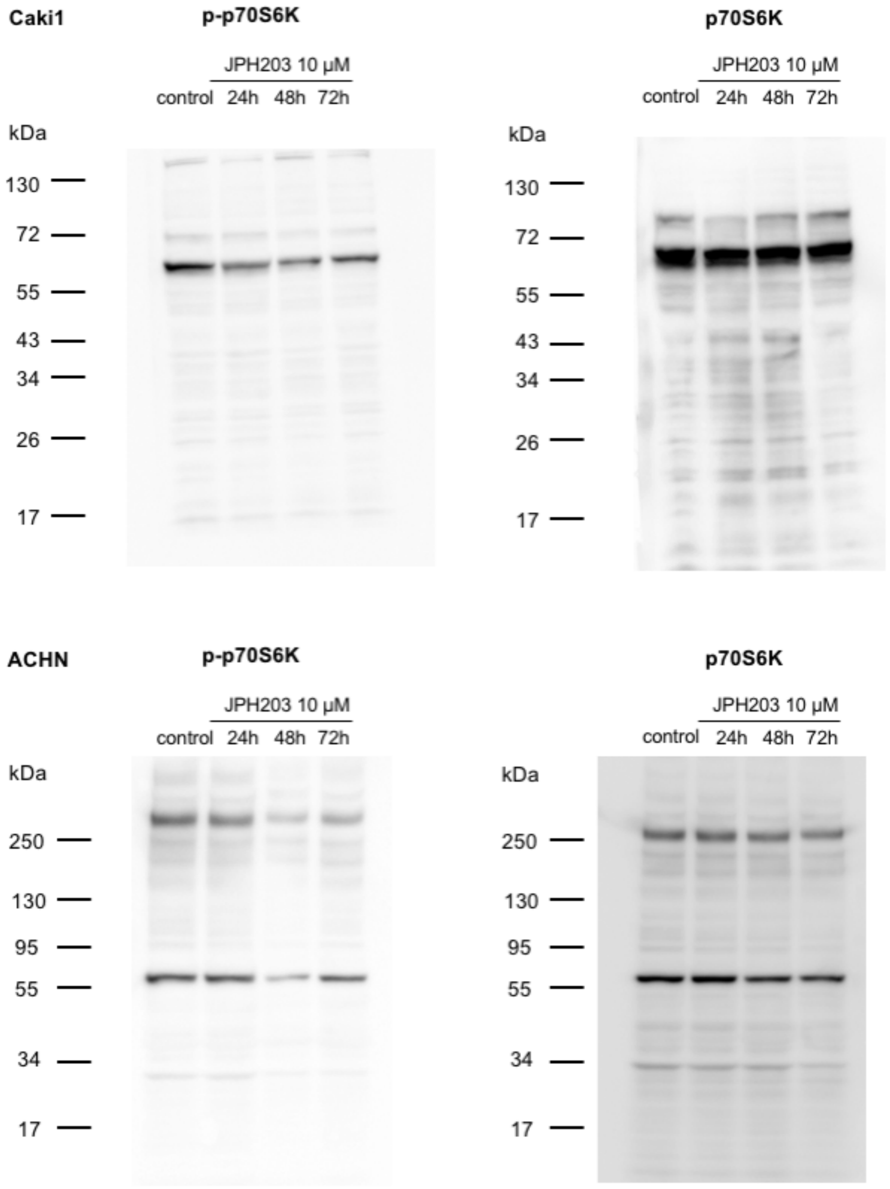

Fig. S8. mTOR and phosphorylated mTOR full-length western blot analysis

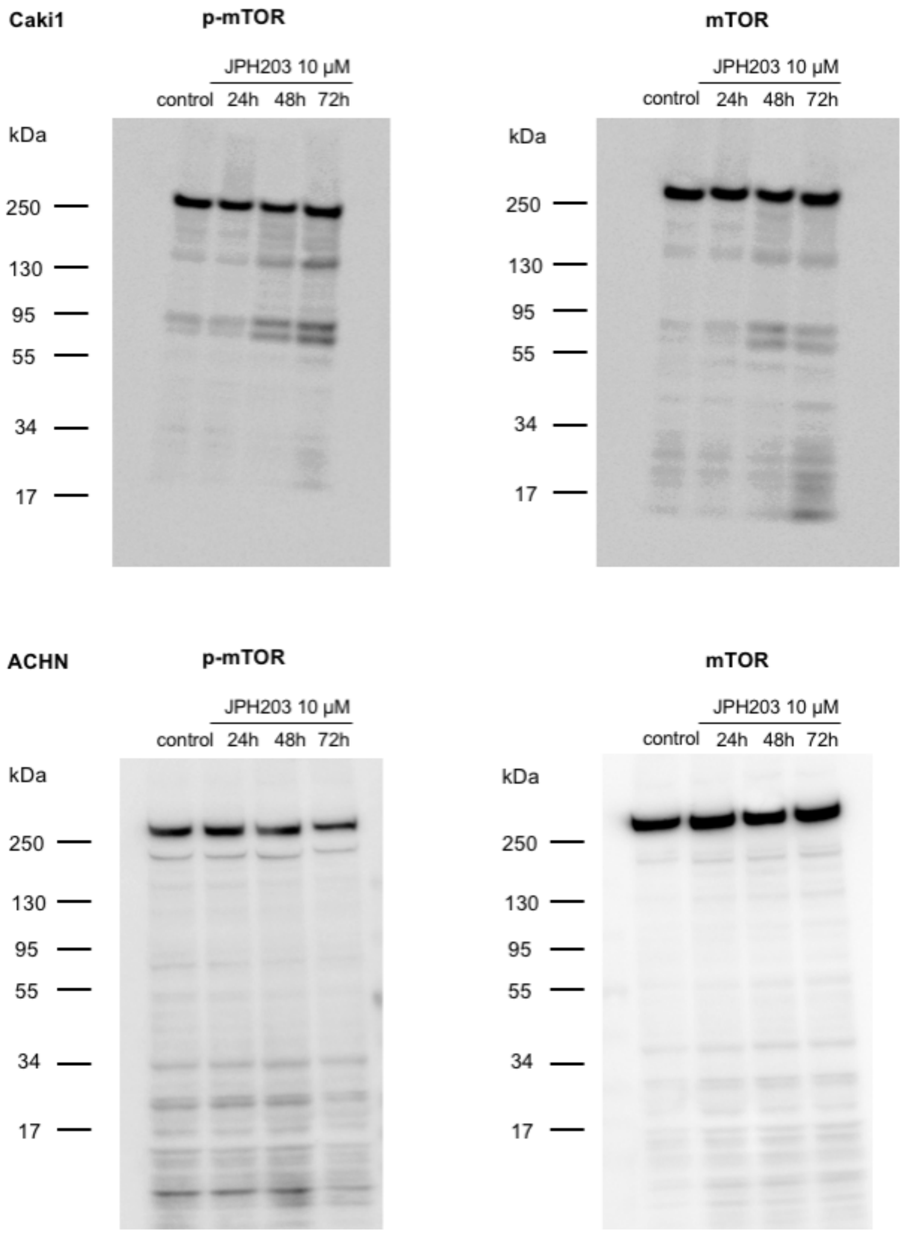

Fig. S9. β-actin full-length western blot analysis

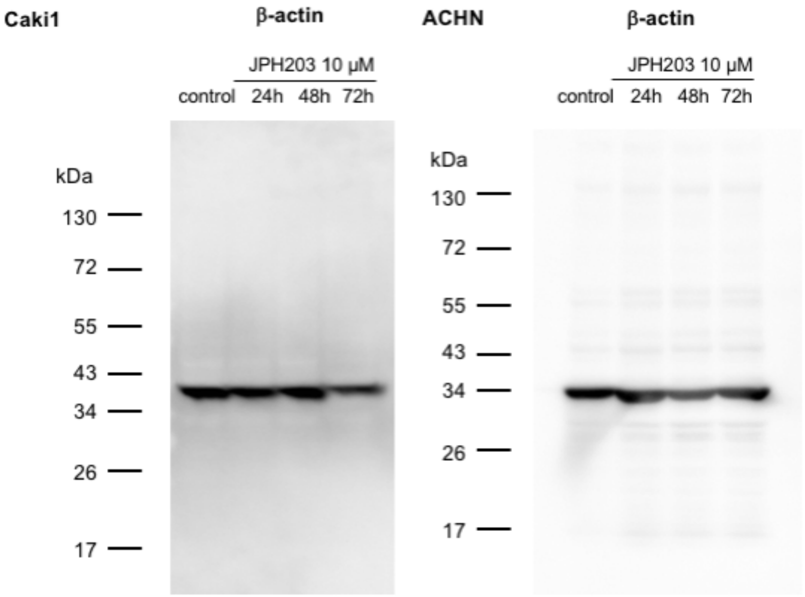

Supplement: Supplementary file 1 — Supplementary information [file 41598_2019_53397_MOESM1_ESM.pdf]
